# β_2_-Adrenergic Ion-Channel Coupled Receptors as Conformational Motion Detectors

**DOI:** 10.1371/journal.pone.0018226

**Published:** 2011-03-25

**Authors:** Lydia N. Caro, Christophe J. Moreau, Jean Revilloud, Michel Vivaudou

**Affiliations:** 1 CNRS, Institut de Biologie Structurale, Grenoble, France; 2 CEA, Institut de Biologie Structurale, Grenoble, France; 3 Université Grenoble I, Institut de Biologie Structurale, Grenoble, France; Cardiff University, United Kingdom

## Abstract

Ion Channel-Coupled Receptors (ICCRs) are artificial proteins comprised of a G protein-coupled receptor and a fused ion channel, engineered to couple channel gating to ligand binding. These novel biological objects have potential use in drug screening and functional characterization, in addition to providing new tools in the synthetic biology repertoire as synthetic K^+^-selective ligand-gated channels. The ICCR concept was previously validated with fusion proteins between the K^+^ channel Kir6.2 and muscarinic M_2_ or dopaminergic D_2_ receptors. Here, we extend the concept to the distinct, longer β_2_-adrenergic receptor which, unlike M_2_ and D_2_ receptors, displayed barely detectable surface expression in our *Xenopus* oocyte expression system and did not couple to Kir6.2 when unmodified. Here, we show that a Kir6.2-binding protein, the N-terminal transmembrane domain of the sulfonylurea receptor, can greatly increase plasma membrane expression of β_2_ constructs. We then demonstrate how engineering of both receptor and channel can produce β_2_-Kir6.2 ICCRs. Specifically, removal of 62–72 residues from the cytoplasmic C-terminus of the receptor was required to enable coupling, suggesting that ligand-dependent conformational changes do not efficiently propagate to the distal C-terminus. Characterization of the β_2_ ICCRs demonstrated that full and partial agonists had the same coupling efficacy, that an inverse agonist had no effect and that the stabilizing mutation E122 W reduced agonist-induced coupling efficacy without affecting affinity. Because the ICCRs are expected to report motions of the receptor C-terminus, these results provide novel insights into the conformational dynamics of the β_2_ receptor.

## Introduction

Ion channel-coupled receptors [ICCRs] are protein-based biosensors created by the covalent assembly of a G Protein-Coupled Receptor [GPCR] and a potassium channel[1]. In such a system, the receptor-channel assembly is engineered to optimize physical interactions between the two proteins so that the conformational changes induced by ligand binding to the receptor are transduced into changes in channel gating, resulting in modification of the recorded ionic current directly correlated with the ligand concentration. These constructs combine the advantages of the two proteins: 1) Ion channels generate electrical signals, large enough to permit single molecule detection; 2) GPCRs recognize chemical ligands with high specificity and affinity. Applications are envisioned in GPCR drug screening by integration in existing ion channel screening platforms or in future microelectronic systems for diagnostic devices or real-time detectors of chemical compounds.

As an initial proof-of-concept, we created functional ICCRs using the inward rectifier K^+^ channel Kir6.2 and two distinct model receptors: the muscarinic receptor M_2_ and the long dopaminergic receptor D_2_ [1]. These ICCRs, designated M_2_-K and D_2_-K, were obtained by fusing receptor C-terminus to channel N-terminus. We demonstrated that receptor-channel coupling could only be achieved after removal of the first 20–25 residues of the channel, without modification of the receptor C-termini. M_2_ and D_2_ receptors are coupled to G_i/o_ proteins and are characterized by short cytoplasmic C-termini. To extend the ICCR concept and examine the impact of a longer receptor C-terminus, we coupled to Kir6.2 a G_s_-protein-coupled receptor with an extended C-terminus, the human β_2_-adrenergic receptor [β_2_AR].

The β_2_AR represents one of the most studied GPCRs. It is involved in smooth muscle (vascular, airway and uterine) relaxation. Because of its physiological role, the β_2_-adrenergic receptor constitutes a target of interest for a wide range of drugs[2]. Indeed, β-blockers are used for treatment of hypertension, glaucoma or after a myocardial infarction[3], while β_2_AR agonists are widely used to treat asthma and premature prenatal contractions. Recently, an engineered β_2_-adrenergic receptor structure was solved at 2.4 Å[4], providing detailed structural information.

The channel protein that we have used to build ICCRs is Kir6.2, the pore-forming subunit of the ATP-sensitive potassium channel (K_ATP_ channel), the other regulatory subunit being the sulfonylurea receptor SUR[5]. The K_ATP_ channel is constituted of 4 Kir6.2 subunits, which form a K^+^-selective pore, and 4 sulfonylurea receptor [SUR] proteins[6]. Within this octameric complex, SUR can modulate the gating of Kir6.2 under the influence of internal adenine nucleotides and pharmacological compounds such as sulfonylureas and K-channel-openers[7], [8]. Kir6.2 is itself directly inhibited by intracellular ATP through a unique nucleotide binding pocket presumably made up of the N-terminal tail of a Kir6.2 subunit and the C-terminal end of the neighbor[9]. This property of Kir6.2 serves as a simple way to identify it and adjust its open probability. Among other SUR regions that interacts with Kir6.2[10], one of the transmembrane domains of SUR, TMD0, is known to tightly bind to Kir6.2 and to facilitate its trafficking to the plasma membrane[11], [12].

Here, we report the successful engineering and characterization of β_2_-based ICCRs. A prerequisite to this project was to find a way to overcome poor surface expression of β_2_AR-Kir6.2 fusion proteins. This was achieved by co-expression of TMD0 of isoform SUR1 which dramatically increased surface expression of all constructs through its interactions with Kir6.2. Pharmacological characterization of β_2_ ICCRs demonstrated concentration-dependent effects of β-adrenergic agonists and antagonists. In addition, the amplitude of the agonist-induced signal depended on the receptor-channel linker length, corroborating previous observations[1] and demonstrating the crucial role of the receptor C-terminus in coupling efficiency. We also examined the effect of a β_2_AR stabilizing mutation at position 3.41 in the Ballesteros/Weinstein scheme[13], [14] on the communication between receptor and channel and found that it logically reduced the amplitude of the agonist responses.

Part of this work has been published in abstract form[15].

## Results

### Design of β2 ICCRs

**Figure 1:**
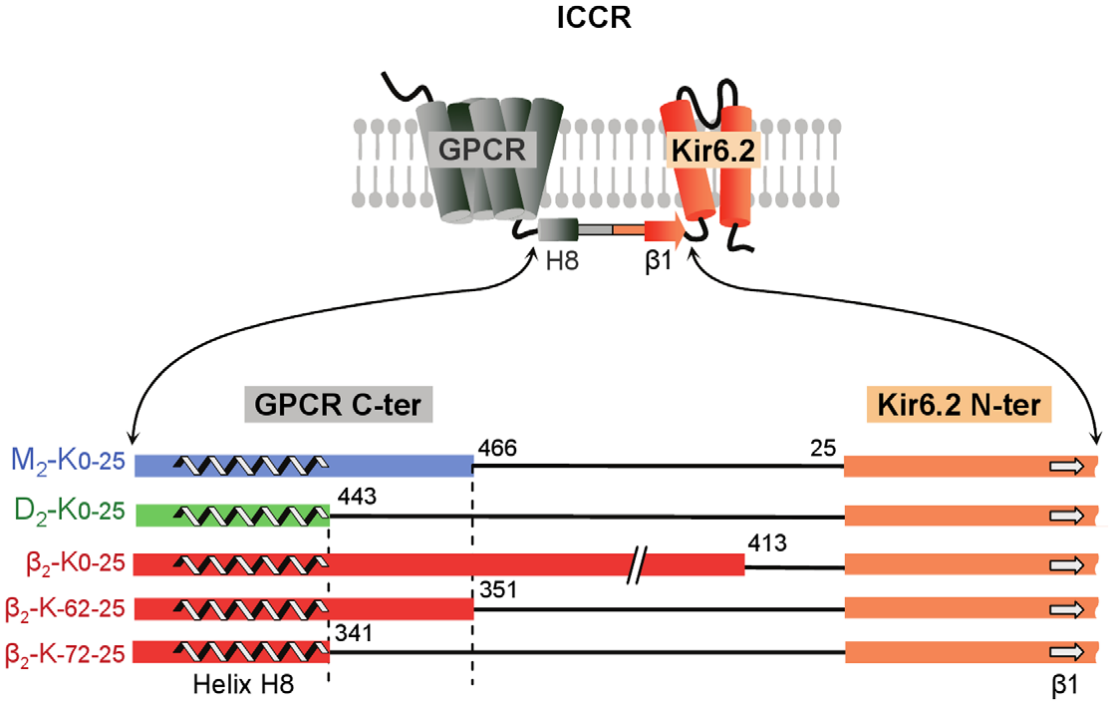
Design strategy of β_2_-based Ion Channel-Coupled Receptors. ICCRs were formed by covalent linkage of GPCRs C-termini to Kir6.2 channel N-terminus. Helix H8 and β-bridge β1 are predicted from the β_2_AR (PDB code: 2RH1) and chimeric Kir3.1 (PDB code: 2QKS) structures, respectively. M_2_-K_0–25_ and D_2_-K_0–25_ are the ICCRs previously shown to be functional with 25 residues deleted from the Kir6.2 N-terminus. We used the same Kir6.2 deletion to build β_2_ ICCRs, with additional deletions in the receptor C-terminus. β_2_-K_0–25_ ICCR contains the full-length receptor, β_2_-K_-62-25_ and β_2_-K_-72-25_ are based on the β_2_AR deleted of 62 and 72 residues in its C-terminal domain to match the lengths of M_2_ and D_2_, respectively.

Building the original M_2_ and D_2_ ICCRs helped delineate the blueprints for other ICCRs. We therefore used the M_2_ and D_2_ ICCRs as templates for expedient design of β_2_ ICCRs. Although M_2_ and D_2_ display a low overall sequence similarity of <30% with β_2_AR, the sequence of the H8 helix is well conserved and was used to unambiguously align the C-terminal extremities of the receptors (Fig. 1). This alignment shows that the C-terminus of β_2_AR is much longer than that of M_2_ and D_2_. Reasoning that this long C-terminus might preclude proper coupling, we constructed three β_2_-based ICCRs: one using the full-length receptor, and two comprised of C­terminal truncated forms of β_2_AR (β_2ΔC62_ and β_2ΔC72_), equivalent in length to the M_2_ and D_2_ receptors, respectively. These receptors were fused to a truncated Kir6.2 lacking its first 25 N-terminal residues, a modification that was shown to produce the most efficient coupling in M_2_ and D_2_ ICCRs[1]. To designate the constructs, we use the nomenclature R-K_-X-Y_, where R is replaced by the short name of the receptor, K stands for Kir6.2, X and Y are the number of residues removed from the receptor C-terminus and channel N-terminus, respectively. The β_2_ constructs are therefore named β_2_-K_0_–_25_, β_2_-K_-62-25_, and β_2_-K_-72-25_.

### Optimizing surface expression

**Figure 2:**
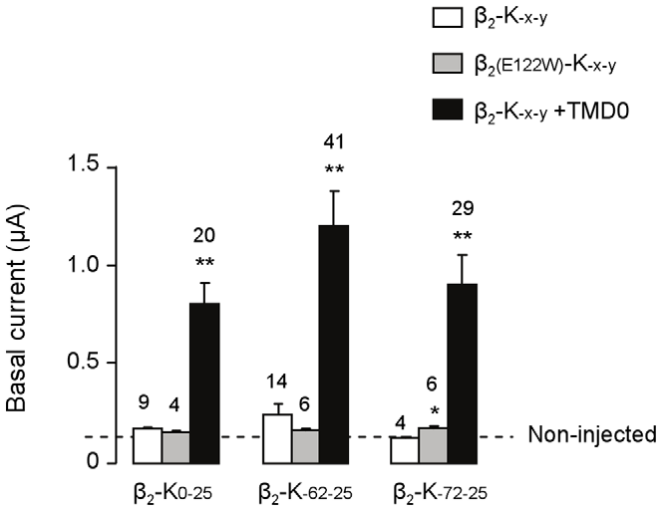
TMD0 of SUR boosts expression of β_2_-based ICCRs. Basal currents are the whole-cell currents measured in the first minute of TEVC recording from unstimulated *Xenopus* oocytes. E122 W is a mutation of residue 122 of β_2_AR from Glu to Trp reported to increase β_2_ surface expression.TMD0 is the first transmembrane domain of the sulfonylurea receptor SUR1, a physiological partner of Kir6.2. *P<0.05 and **P<0.00001 represent significant differences from the basal current measured in non-injected oocytes.

Constructs were expressed in *Xenopus* oocytes and characterized by the two-electrode voltage clamp technique. As a rough estimate of surface expression levels, we measured the basal currents, i.e., the initial whole-cell currents, (Fig. 2). The three β_2_ constructs produced basal currents that were equivalent to those obtained with non-injected oocytes suggesting no or little expression of active channels. In an attempt to solve this expression problem, we engineered ICCRs using the β_2(E122__W)_ mutant. This mutation of Glu_122_ to Trp_122_ at Ballesteros/Weinstein position 3.41[13] has been shown to enhance the surface expression level of the β_2_-adrenergic receptor in insect and mammalian cells by stabilizing the TM4-TM3-TM5 helix interface[14]. This mutation had no effect on the basal current of β_2_-based ICCRs.

It has been demonstrated that N-terminal deletions could favor cell surface expression of the cannabinoid receptor 1[16] and the α_1D_-adrenergic receptor[17]. We therefore tried gradual N-terminal deletions of the first 10 to 25 residues of β_2_AR in construction β_2_-K_-62-25_. The data shown in Fig. S1 show that these modifications did not improve expression. Also shown in Fig. S1 are the disappointing outcomes of using N-terminal and C-terminal chimera between β_2_AR and the robustly-expressed M_2_ receptor.

We then tested the co-expression of TMD0, a 195-residue N-terminal transmembrane domain of SUR1, known to facilitate Kir6.2 trafficking[11], with β_2_-based ICCRs. The resulting basal current was increased 5-fold for β_2_-K_0–25_ and β_2_-K_-62-25_ and 7-fold for β_2_-K_-72-25_ compared to the ICCRs expressed alone. Thus, we found an efficient way to enhance significantly surface expression levels of the β_2_-based ICCRs. These results suggest that TMD0 helps the β_2_-based ICCRs reach the membrane because of its chaperone role on Kir6.2.

### Demonstration of direct receptor-channel coupling

**Figure 3:**
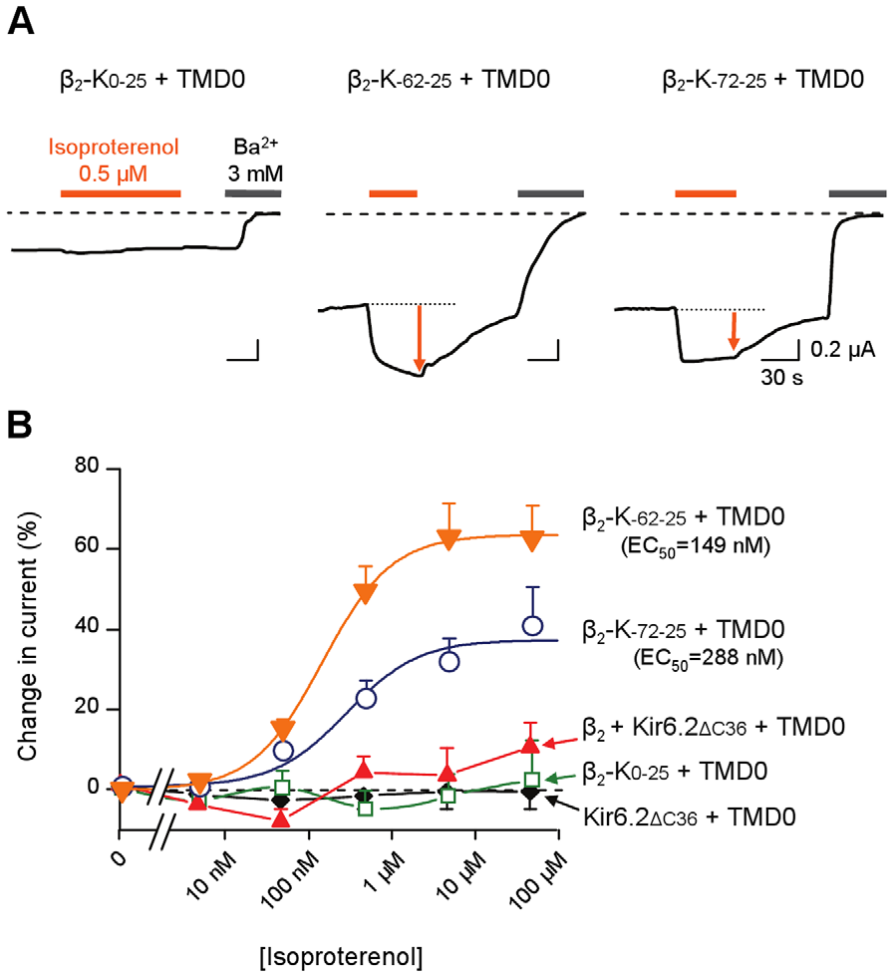
Receptor-channel coupling in β_2_ ICCRs: response to the agonist isoproterenol. (*A*) Representative TEVC recordings from *Xenopus* oocytes expressing each β_2_ ICCR and TMD0. Membrane potential was −50 mV. Dashed lines indicate the baseline of Ba^2+^-sensitive currents. (*B*) Concentration-effect curves for isoproterenol measured in oocytes co-expressing the indicated proteins. Kir6.2_ΔC36_ is deleted of its last 36 residues to allow surface expression of the channel alone. Values are average of 5–14 measurements. Smooth lines correspond to Hill equations fits with EC_50_ in parentheses and h = 1.07 for β_2_-K_-62-25_ and 1 for β_2_-K_-72-25_.

The functionality of the coupling between β_2_AR (full-length, ΔC62, ΔC72) and Kir6.2 was tested with the β-adrenergic agonist isoproterenol. We initially verified that isoproterenol had no direct or receptor-mediated effects on Kir6.2 alone or co-expressed with β_2_AR (Fig. 3B). When the fusion proteins where expressed (with TMD0), β_2_-K_0–25_ did not respond to isoproterenol whereas β_2_-K_-62-25_ and β_2_-K_-72-25_ were strongly activated (Fig. 3). Isoproterenol responses were concentration-dependent with no obvious cooperativity (Hill coefficients ∼1). Given the variability in the data, the EC_50_ of 149 nM for β_2_-K_-62-25_ and 288 nM for β_2_-K_-72-25_ were not significantly different (p = 0.31; unpaired Student's t-test). These values are consistent with those from other techniques that do not rely on G-protein signalling such as competitive radioligand binding or fluorescence spectroscopy[18], [19]. The maximal channel activation was 64% of the basal current for β_2_-K_-62-25_ and 37% for β_2_-K_-72-25_, a statistically significant difference (p = 0.018). This change in efficacy without change in affinity underscores the role of the length of the receptor-channel linker region in efficient transmission of the ligand-induced β_2_ conformational change to the channel gate.

**Figure 4:**
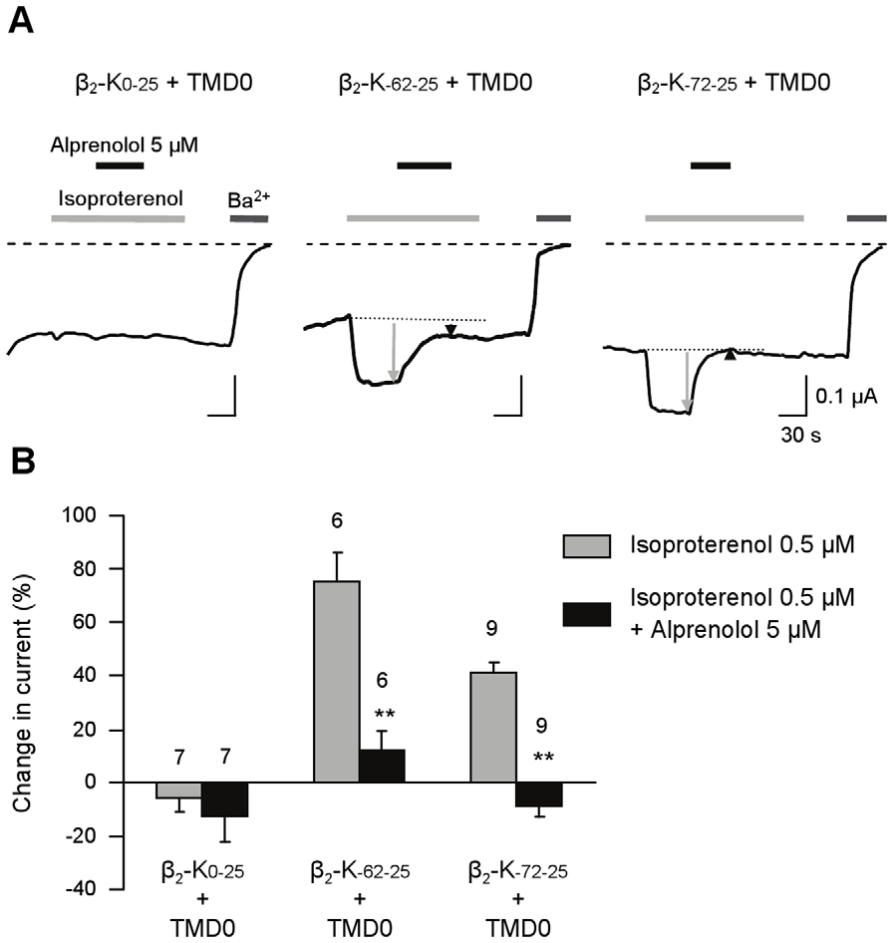
Effect of a β-adrenergic antagonist on β_2_ ICCRs. (*A*) TEVC recordings showing antagonist effect of 5 µM alprenolol during addition of 0.5 µM isoproterenol on β_2_-K_0–25_, β_2_-K_-62-25_ and β_2_-K_-72-25_. (*B*) Change in whole-cell currents evoked by isoproterenol before and after addition of 5 µM alprenolol. **P<0.00075 indicates a significant inhibition induced by alprenolol.

We next tested the effect of the antagonist alprenolol at 5 µM on the isoproterenol-activated current. Alprenolol did not alter the current generated by the isoproterenol-insensitive construct β_2_-K_0–25_ but it caused a complete block of isoproterenol activation of β_2_-K_-62-25_ and β_2_-K_-72-25_ (Fig. 4). This block could not be washed out after several minutes, probably because we used a relatively high alprenolol concentration. These results confirmed the specificity of isoproterenol effect on the β_2_ adrenergic receptor.

### Partial and inverse agonists

**Figure 5:**
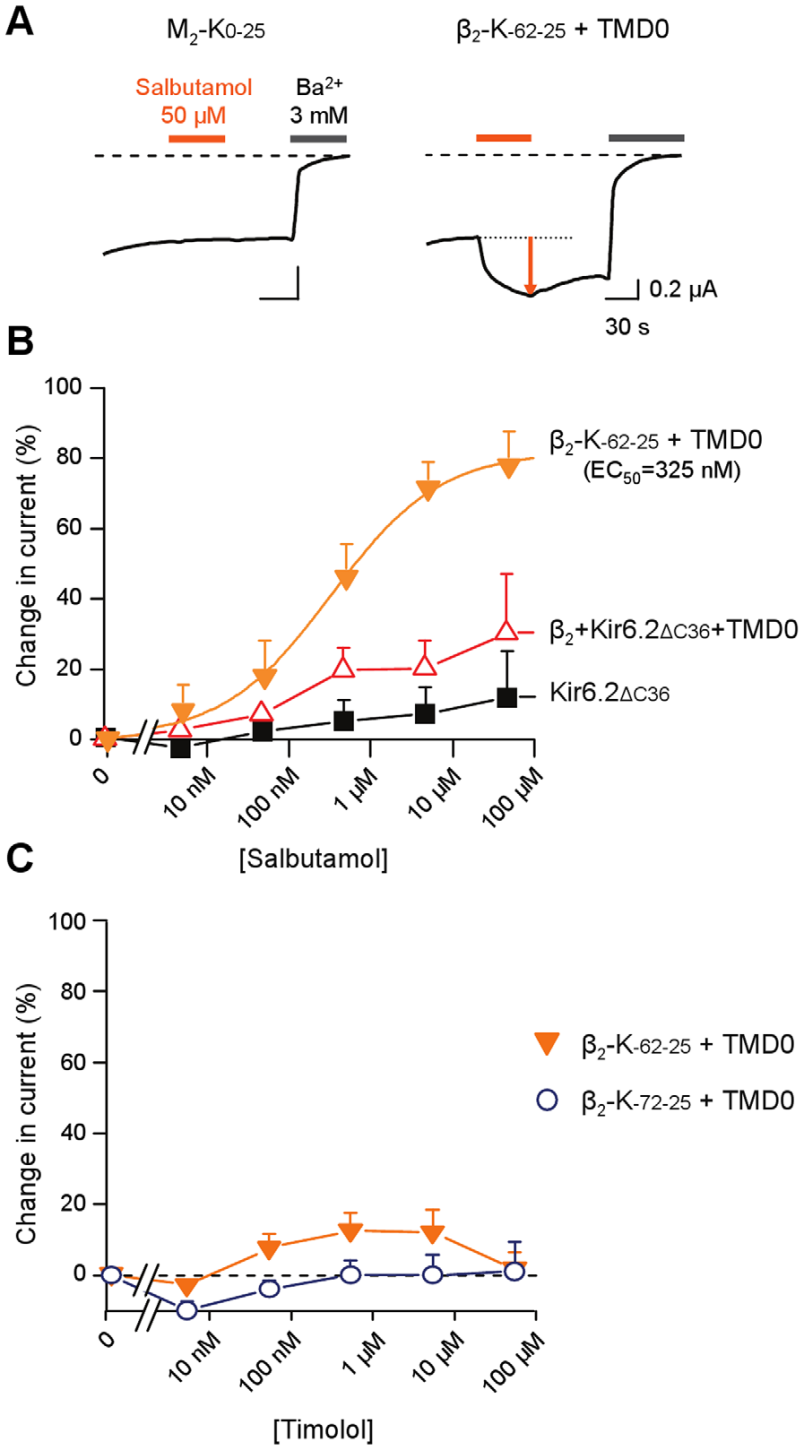
Effect of a β_2_AR partial agonist on β_2_-K_-62-25_. Concentration-effect curves for salbutamol measured in oocytes co-expressing the indicated proteins. Values are average of 3-7 measurements. The smooth line is a Hill equation fit to the β_2_-K_-62-25_+TMD0 data with EC_50_  =  452 nM and h = 0.6. Data obtained with the unfused Kir6.2 as a control could not be fitted.

Full agonists can cause maximal activation of the receptor whereas partial agonists cause an activation which remains less than maximal even at saturating concentrations. It is thought that full and partial agonists of the β-adrenoceptor do not trigger the same conformational changes in the receptor. As a comparison with the full agonist isoproterenol, we therefore assayed the partial agonist salbutamol on construct β_2_-K_-62-25_. As shown in Fig. 5, salbutamol strongly activated β_2_-K_-62-25_. The maximal activation, 78% of the basal current at 50 µM, was larger, though not significantly (p = 0.12) than that achieved by isoproterenol, 63% at 50 µM. The concentration dependence was not as steep with a Hill slope of 0.64 compared to 1.04 for isoproterenol. Although these differences remain rather subtle, they reinforce the notion of distinct modes of action for partial and full agonists[20].

Inverse agonists, thought to target the agonist binding site, downregulates the receptor by blocking its constitutive activity. We examined the effects of the inverse agonist timolol[21]. At concentrations up to 50 µM, timolol did not produce any significant change in the electrical signal from either β_2_-K_-62-25_ or β_2_-K_-72-25_ (Fig. 5C). This lack of effects suggests that binding of timolol does not induce a large conformational change in the receptor.

Another possibility could be that the ICCRs are partly cleaved and that we could have an unresponsive Kir6.2 breakdown product responsible for the high basal current together with a responsive full-length fusion construct with no basal current. In that case, an already inactive construct could not possibly be further inhibited by timolol. This hypothesis is highly improbable because 1) we have never detected any breakdown products by Western blot in other similar fusion constructs not included in the present work, and 2) Fig. S2 shows that Kir6.2 + TMD0 produces a basal current that is barely detectable.

### A stabilizing mutation alters coupling

**Figure 6:**
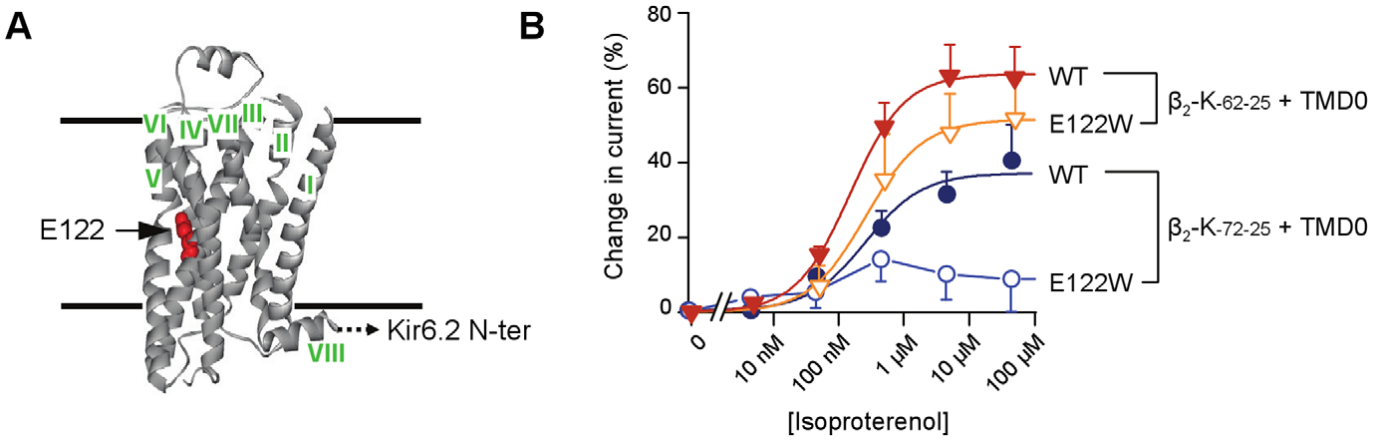
The stabilizing mutation E122 W weakens agonist-induced channel responses. (*A*) Location of Glu_122_ (in red) in the β_2_-adrenergic receptor structure. (*B*) Concentration-effect curves of isoproterenol on β_2_-K_-62-25_ and β_2_-K_-72-25_, unmodified (WT) and harboring mutation E122 W (all co-expressed with TMD0). Values are average of 5–14 measurements. Hill equation fits, represented as smooth lines, yielded EC_50_ of 149 nM, 247 nM, and 288 nM for β_2_-K_-62-25_, β_2_(E122 W)-K_-62-25_, and β_2_-K_-72-25_, respectively. h was 1.07, 1, and 1.18.

To further show that ICCR systems can be used as a functional characterization tool, we set out a study on the E122 W β_2_AR mutant described above. As described in Fig. 6, for construct β_2_-K_-62-25_, mutation E122 W appeared to reduce the amplitude of the agonist-induced signal (from 63.5% to 51% at maximum activation) and to increase dissociation constant (from 149 nM to 247 nM) but these effects did not reach statistical significance (p = 0.12 for amplitudes; p = 0.24 for affinities). The mutation had a stronger effect on construct β_2_-K_-72-25_ since maximal activation decreased from 37% to 11%, a statistically significant change (p = 0.017). In that case, affinities could not be compared because the activation of the β_2(E122__W)_-K_-72-25_ was too weak for proper fitting. These results could be explained by the fact that stabilization of the TM4-TM3-TM5 helix interface[14] induces less important conformational change in β_2_AR upon ligand binding. Indeed, position 3.41 is located at the TM4-TM3-TM5 interface and the Trp ring may interact with Pro211^5.50^ partially decreasing TM5 flexibility. Since TM5 is assumed to serve as an intermediate between the TM1-4 structural core and TM6-7[22], we can imagine that the quality of the transmission of conformational change may be constrained by such mutation. This might lead to a less efficient communication with Kir6.2 resulting in decreased amplitude of the response.

## Discussion

Using the ICCR concept established with M_2_ and D_2_ receptors[1], we have used the β_2_-adrenergic receptor to create synthetic ligand-gated K^+^ channels sensitive to β-adrenergic ligands.

### Surface expression enhancement by an accessory Kir6.2-binding protein

A recurrent difficulty with recombinant membrane proteins is the low density of proteins that reach the plasma membrane. Although *Xenopus* oocytes are very tolerant in that respect, expression of the β_2_-Kir6.2 fusion constructs produced no discernible electrophysiological signals. Suspecting a trafficking impediment, we searched for ways to enhance surface expression. It is known that Kir6.2 possess a C-terminal endoplasmic-reticulum retention signal[23] but removal of this signal in M_2_ ICCRs did not augment surface expression[1]. The mutation E122 W in β_2_AR, reported to increase surface expression[14] was also not beneficial. The solution came from the K_ATP_ channel. That channel is a complex of Kir6.2 and the protein SUR. Association of SUR to Kir6.2 is known to be mediated in large part by its N-terminal transmembrane domain TMD0, a ∼200-residue alpha-helical region that binds to Kir6.2 by itself and can promote its targeting to the surface membrane[11]. When the TMD0 domain of the sulfonylurea receptor isoform SUR1 was co-expressed with the various β_2_-Kir6.2 constructs, large K^+^ currents could be recorded indicative of the presence of active Kir6.2 at the oocyte surface. This discovery was the key to the pursuit of the project. It suggests that, in the tetrameric β_2_-Kir6.2 complexes, there is ample space for TMD0 to bind to Kir6.2 and to act as a chaperone to promote proper membrane targeting.

### Functional β2 ICCRs

The β_2_ ICCRs were engineered by covalent linkage of β_2_AR to the Kir6.2 channel to promote physical interactions between the two proteins. Functional coupling could only be achieved after removal of 25 residues from the Kir6.2 N-terminus, as in previous ICCRs, and also of 62 to 72 residues from the β_2_AR C-terminus whereas M_2_ and D_2_ ICCRs used unmodified receptors. These residues which are not resolved in crystallographic structures[4] probably form flexible elements[24] that dampen transmission of mechanical perturbations from receptor to channel. The dependence of responses on the length of the receptor-channel argues strongly for a direct, physical interaction between receptor and channel. We also verified the lack of detectable G-protein dependent modulation of Kir6.2 by β_2_AR in control experiments where receptor and channel were coexpressed as separate proteins. Furthermore, β_2_AR is predominantly G_s_-coupled, the M_2_ receptor is G_i_-coupled, but both produce similar effects when fused to Kir6.2.

### ICCRs as conformational motion detectors

Constructs β_2_-K_-62-25_ and β_2_-K_-72-25_ (+TMD0) detected the presence of agonists with dose-dependent correlation, in direct, real-time and label-free conditions. The affinity measured for the full agonist isoproterenol matched those obtained by radioligand assays[14], [19] as well as spectroscopy assays that, like ICCRs, directly measure conformational changes[18]. The effect of the partial agonist salbutamol was similar to that of isoproterenol although it showed lower affinity as expected. Isoproterenol and salbutamol have been shown to induce distinct conformations. In particular, evidence suggests that both disrupt the cytoplasmic ionic lock while only isoproterenol uses the rotamer toggle switch[25]. The similarity of the responses elicited by salbutamol and isoproterenol suggests that the conformational changes detected by the channel could be related to the ionic lock rather than the rotamer toggle switch[25]. Because by construction ICCRs report on the motion of the GPCR C-terminus, this would imply that disruption of the ionic lock triggers a conformational change in the C-terminus.

The effect of the antagonist alprenolol was easily detectable by abolition of the agonist-induced increase of the ionic current. If alprenolol did not change basal signal, inverse agonists are expected to reduce basal activity and elicit signals in absence of agonists. In the ICCR assay, the inverse agonist timolol produced no significant signal. Although this observation could result from an intrinsically low basal activity of β_2_AR due to the expression system or the fusion to Kir6.2, it shows that binding of timolol does not induce any detectable conformational change of the C-terminus. Such conclusion is consistent with a recent crystallographic study[26] showing only very small differences between the antagonist-bound and inverse-agonist-bound structures of β_2_AR.

Thus, beside the obvious use of ICCRs in drug screening, they could be valuable to dissect the conformational changes induced by ligands. We provided an additional example of such use by demonstrating that a stabilizing mutation, E122 W_3.41_ [14], reduced the amplitude of the ICCR response in line with its purported attenuation of conformational changes.

### Physiological relevance

ICCRs demonstrate that, provided a GPCR is tightly associated with an ion channel, it can directly modulate channel gating possibly through mechanical forces transmitted by its C-terminal tail. Did evolution overlook this seemingly trivial possibility of using localized modulation in addition to the more indiscriminate second-messenger pathways? Probably not, as there is solid evidence that receptors and channels can form stable complex[27]–[29]. Channel modulation via the C-terminal tail of GPCRs has been reported for 2 couples, GABA_A_ channel/dopamine D_5_ receptor[30] and NMDA channel/dopamine D_1_ receptor[31]. ICCRs could provide a model for these interactions as well as for others involving Kir channels[32].

### β-adrenergic ligand-activated K^+^ channels

Like traditional ligand-gated channels such as the cationic nicotinic acetylcholine receptor or the anionic GABA_A_ receptor[33], β_2_ ICCRs incorporates in a single polypeptide chain a binding site for a specific signaling molecule and an ion-selective pore that are allosterically linked. They possess, however, the unique features among ligand-gated channels of being activated by β-adrenergic signals and of being selective for potassium ions. One may envision that these ICCRs could be used as novel regulatory elements in synthetic biology as well as therapeutic tools. Such use is of course remote and would require to augment trafficking efficiency to avoid using accessory proteins such as TMD0 and optimize response efficacy so that channels are closed at rest and open upon stimulation like existing ligand-gated channels. This would require protein engineering that is now complex but could become more straightforward as determinants of membrane protein trafficking and of channel gating are clarified.

## Materials and Methods

### Molecular biology

Experiments were conducted as previously described[1]. In this work, we used mouse Kir6.2 (Genbank D50581)[34], human β_2_-adrenergic receptor (Genbank NM_000024.3), hamster TMD0(SUR1)-F195[11], [35], mouse Kir6.2_ΔC36_ [36]. The β_2_-K_0–25_ fusion was obtained by replacing the muscarinic M_2_ receptor gene in M_2_-K_0–25_ cloned in the *Xenopus* oocyte expression vector pGEMHE[1]. Insertion of the β_2_AR gene and deletion of the M_2_ gene was performed using a two-step PCR. In the first PCR reaction, the β_2_-adrenergic gene was amplified from its original pCMV vector using hybrid primers complementary to the β_2_-adrenergic sequence 3′ extremities and to the flanking regions of the insertion site in the M_2_-Kir6.2_ pGEMHE. The products of this reaction were gel-purified (QIAquick Gel Extraction Kit, Qiagen) and served as primers for a second PCR with M_2_-K_0–25_ as a template, yielding β_2_-K_0–25__pGEMHE. Alignments of the M_2_, D_2_, and β_2_ receptor sequences with ClustalX[37] were adjusted manually to position conserved helix H8. The unstructured C-terminal region downstream of H8 was longer in the β_2_AR by 62 and 72 amino acids compared to M_2_ and D_2_, respectively (Fig. 1). To match the lengths of M_2_ and D_2_, additional β_2_-K constructs with shorter β_2_AR C-termini were obtained in a single-step PCR using the β_2_-K_0–25_ construct as a template and hybrid oligonucleotides flanking the deleted region[38]. Mutation E122 W was introduced in each ICCR in a single-step PCR with oligonucleotides incorporating the mutation. Reagents and conditions were from the QuikChange site-directed mutagenesis kit (Agilent Technologies). Positive clones were identified by restriction enzyme profiling and verified by sequencing the full open reading frame.

After DNA amplification, constructs were linearized and mRNAs synthesized using the T7 mMessage mMachine Kit (Ambion). mRNAs were purified either by standard phenol:chloroform extraction or using the MEGAclear Purification Kit (Ambion), and quantified by agarose-gel electrophoresis and spectrophotometry.

### Electrophysiological recordings

Animal handling and experiments fully conformed with French regulations and were approved by local governmental veterinary services (authorization no. 38-08-10 from the Ministère de l'Agriculture, Direction des Services Vétérinaires to Michel Vivaudou). Oocytes were surgically removed from *Xenopus laevis* and defolliculated by three 30 min-incubations in 2 mg.ml^−1^ type 1A collagenase solution at 19°C. Stage V and VI oocytes were microinjected with 50 nl of RNase-free water containing one or a mixture of the following quantities of RNA: β_2_-Kir6.2, 5 ng; Kir6.2_ΔC36_, 2 ng; TMD0(SUR1)-F195, 1 ng. Microinjected oocytes were incubated for >2 days at 19°C in Barth's solution (in mM: 1 KCl, 0.82 MgSO_4_, 88 NaCl, 2.4 NaHCO_3_, 0.41 CaCl_2_, 16 Hepes, pH 7.4) supplemented with 100 U.ml^−1^ penicillin, streptomycin and gentamycin. All chemicals were purchased from Sigma-Aldrich. Whole-cell currents were recorded with the two-electrode voltage clamp (TEVC) technique using a GeneClamp 500 amplifier (Molecular Devices). Microelectrodes were filled with 3 M KCl and oocytes were bathed in the following solution (in mM): 91 KCl, 1.8 CaCl_2_, 1 MgCl_2_, 5 HEPES, 0.3 niflumic acid (to block endogenous Cl^-^ currents), pH 7.4. The TEVC voltage protocol consisted of 500-ms steps to -50, 0 and +50 mV – during which current was measured – separated by 5 s at a holding potential of 0 mV. The values shown in the figures are those recorded at −50 mV.

### Data analysis

Basal current was measured while oocytes were in standard bath solution during the first minute of recording. Ba^2+^ (3 mM) was used as a generic potassium-channel blocker to establish the amount of exogenous current, designated as Ba^2+^-sensitive current and calculated by subtracting from all measured values the value measured at the end of an experiment after application of 3 mM Ba^2+^. All values of current reported here refer to Ba^2+^-sensitive currents. Changes in Ba^2+^-sensitive currents by effectors were calculated with respect to the value measured before application. The points at which the current were measured on the current traces are indicated by arrows in the figures. For the concentration-response data, obtained by sequential application of increasing agonist concentrations, changes in current were calculated only with respect to the current before application of the initial, lowest concentration.

Average values are presented as mean±s.e.m. Non-linear least-square curve-fitting was carried out with Origin 8 software (OriginLab) using a standard Hill equation:

f(x) = Max/[1 + (EC_50_/x)^h^]

where x is the concentration of a ligand, Max the asymptotical maximal effect, EC_50_ the concentration for half-maximal effect, and h the Hill coefficient. The fits shown in the figures were performed using average data. For statistical analysis of parameters Max and EC_50_ (using Origin 8 software), individual dose-response data from each oocyte tested were fitted using the above equation with h = 1 to obtain a set of values of Max and EC_50_ for each construct and ligand. Statistical significance for these parameters and for other experimental data was established with unpaired two-tailed Student t-tests and is indicated as p-values in the text.

## Supporting Information

Figure S1 Expression levels of various β2-K-62-25 constructs designed in an attempt to improve surface expression. The basal currents, whole-oocyte currents recorded in absence of agonist are taken as an indicator of the number of active channels at the cell surface. ΔN10, ΔN15, ΔN20, and ΔN25 designate constructs based on β2-K-62-25 with the first N-terminal 10, 15, 20, and 25 residues of β2AR deleted. Nt(M2)ΔN28 is a β2-K-62-25 chimera where the extracellular N-terminal of β2AR (28 residues) has been replaced by that of the M2 receptor (18 residues). Ct(M2) is a β2-K-62-25 where the intracellular C-terminal of β2ARΔC62 (residues 326 to 352) has been replaced by that of the M2 receptor (residues 440 to 466).
(PDF)

Figure S2 Comparison of the expression levels of Kir6.2, alone or fused to β2, coexpressed with TMD0. The basal currents, whole-oocyte currents recorded in absence of agonist are taken as an indicator of the number of active channels at the cell surface.
(DOC)
