# Supplementary material for: β2-Adrenergic Ion-Channel Coupled Receptors as Conformational Motion Detectors
Source: PLoS One. 2011 Mar 25;6(3):e18226. doi: 10.1371/journal.pone.0018226 (PMC3064670; doi:10.1371/journal.pone.0018226)
Supplement: Figure S1 — Expression levels of various β2-K-62-25 constructs designed in an attempt to improve surface expression. The basal currents, whole-oocyte currents recorded in absence of agonist are taken as an indicator of the number of active channels at the cell surface. ΔN10, ΔN15, ΔN20, and ΔN25 designate constructs based on β2-K-62-25 with the first N-terminal 10, 15, 20, and 25 residues of β2AR deleted. Nt(M2)ΔN28 is a β2-K-62-25 chimera where the extracellular N-terminal of β2AR (28 residues) has been replaced by that of the M2 receptor (18 residues). Ct(M2) is a β2-K-62-25 where the intracellular C-terminal of β2ARΔC62 (residues 326 to 352) has been replaced by that of the M2 receptor (residues 440 to 466). (PDF) [file pone.0018226.s001.pdf]

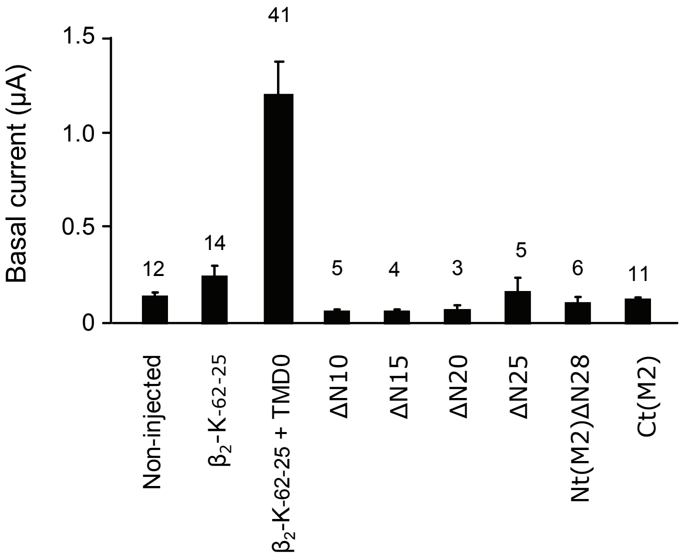

Fig. S1. Expression levels of various β_2-K_{-62-25} constructs designed in an attempt to improve surface expression. The basal currents, whole-oocyte currents recorded in absence of agonist are taken as an indicator of the number of active channels at the cell surface. ΔN10, ΔN15, ΔN20, and ΔN25 designate constructs based on β_2-K_{-62-25} with the first N-terminal 10, 15, 20, and 25 residues of β_2AR deleted. Nt(M2)ΔN28 is a β_2-K_{-62-25} chimera where the extracellular N-terminal of β_2AR (28 residues) has been replaced by that of the M_2 receptor (18 residues). Ct(M2) is a β_2-K_{-62-25} where the intracellular C-terminal of $\beta_2AR_{\Delta C62}$ (residues 326 to 352) has been replaced by that of the M_2 receptor (residues 440 to 466).
