# Supplementary material for: β2-Adrenergic Ion-Channel Coupled Receptors as Conformational Motion Detectors
Source: PLoS One. 2011 Mar 25;6(3):e18226. doi: 10.1371/journal.pone.0018226 (PMC3064670; doi:10.1371/journal.pone.0018226)
Supplement: Figure S2 — Comparison of the expression levels of Kir6.2, alone or fused to β2, coexpressed with TMD0. The basal currents, whole-oocyte currents recorded in absence of agonist are taken as an indicator of the number of active channels at the cell surface. (DOC) [file pone.0018226.s002.doc]

# β2-adrenergic Ion-Channel Coupled Receptors as Conformational Motion Detectors

**Lydia N. Caro1,2,3, Christophe J. Moreau1,2,3, Jean Revilloud1,2,3 and Michel Vivaudou1,2,3***

**1** CNRS, Institut de Biologie Structurale, Grenoble, France, **2** CEA, Institut de Biologie Structurale, Grenoble, France **3** Université Grenoble I, Institut de Biologie Structurale, Grenoble, France

Running head: *β2-adrenergic Ion-Channel Coupled Receptors*

*****Address correspondence to: Michel Vivaudou,IBS, 41 rue Jules Horowitz, 38027 Grenoble, France. Tel: +33-4 38 78 48 67; Fax: +33-4 38 78 54 94; E-mail: vivaudou@ibs.fr

## Abstract

Ion Channel-Coupled Receptors (ICCRs) are artificial proteins comprised of a G protein-coupled receptor and a fused ion channel, engineered to couple channel gating to ligand binding. These novel biological objects have potential use in drug screening and functional characterization, in addition to providing new tools in the synthetic biology repertoire as synthetic K+-selective ligand-gated channels. The ICCR concept was previously validated with fusion proteins between the K+ channel Kir6.2 and muscarinic M2 or dopaminergic D2 receptors. Here, we extend the concept to the distinct, longer β2-adrenergic receptor which, unlike M2 and D2 receptors, displayed barely detectable surface expression in our *Xenopus* oocyte expression system and did not couple to Kir6.2 when unmodified.

Here, we show that a Kir6.2-binding protein, the N-terminal transmembrane domain of the sulfonylurea receptor, can greatly increase plasma membrane expression of β2 constructs. We then demonstrate how engineering of both receptor and channel can produce β2‑Kir6.2 ICCRs. Specifically, removal of 62-72 residues from the cytoplasmic C-terminus of the receptor was required to enable coupling, suggesting that ligand-dependent conformational changes do not efficiently propagate to the distal C-terminus.

Characterization of the β2 ICCRs demonstrated that full and partial agonists had the same coupling efficacy, that an inverse agonist had no effect and that the stabilizing mutation E122W reduced agonist-induced coupling efficacy without affecting affinity. Because the ICCRs are expected to report motions of the receptor C-terminus, these results provide novel insights into the conformational dynamics of the β2 receptor.

### Introduction

Ion channel-coupled receptors [ICCRs] are protein-based biosensors created by the covalent assembly of a G Protein-Coupled Receptor [GPCR] and a potassium channel[1]. In such a system, the receptor-channel assembly is engineered to optimize physical interactions between the two proteins so that the conformational changes induced by ligand binding to the receptor are transduced into changes in channel gating, resulting in modification of the recorded ionic current directly correlated with the ligand concentration. These constructs combine the advantages of the two proteins: 1) Ion channels generate electrical signals, large enough to permit single molecule detection; 2) GPCRs recognize chemical ligands with high specificity and affinity. Applications are envisioned in GPCR drug screening by integration in existing ion channel screening platforms or in future microelectronic systems for diagnostic devices or real-time detectors of chemical compounds.

As an initial proof-of-concept, we created functional ICCRs using the inward rectifier K+channel Kir6.2 and two distinct model receptors: the muscarinic receptor M2 and the long dopaminergic receptor D2[1]. These ICCRs, designated M2-K and D2-K, were obtained by fusing receptor C-terminus to channel N-terminus. We demonstrated that receptor-channel coupling could only be achieved after removal of the first 20-25 residues of the channel, without modification of the receptor C-termini. M2 and D2 receptors are coupled to Gi/o proteins and are characterized by short cytoplasmic C-termini. To extend the ICCR concept and examine the impact of a longer receptor C-terminus, we coupled to Kir6.2 a Gs-protein-coupled receptor with an extended C-terminus, the human β2-adrenergic receptor [β2AR].

The β2AR represents one of the most studied GPCRs. It is involved in smooth muscle (vascular, airway and uterine) relaxation. Because of its physiological role, the β2-adrenergic receptor constitutes a target of interest for a wide range of drugs[2]. Indeed, β-blockers are used for treatment of hypertension, glaucoma or after a myocardial infarction[3], while β2AR agonists are widely used to treat asthma and premature prenatal contractions. Recently, an engineered β2-adrenergic receptor structure was solved at 2.4 Å[4], providing detailed structural information.

The channel protein that we have used to build ICCRs is Kir6.2, the pore-forming subunit of the ATP-sensitive potassium channel (KATP channel), the other regulatory subunit being the sulfonylurea receptor SUR[5]. The KATP channel is constituted of 4 Kir6.2 subunits, which form a K+-selective pore, and 4 sulfonylurea receptor [SUR] proteins[6]. Within this octameric complex, SUR can modulate the gating of Kir6.2 under the influence of internal adenine nucleotides and pharmacological compounds such as sulfonylureas and K-channel-openers[7,8]. Kir6.2 is itself directly inhibited by intracellular ATP through a unique nucleotide binding pocket presumably made up of the N-terminal tail of a Kir6.2 subunit and the C-terminal end of the neighbor[9]. This property of Kir6.2 serves as a simple way to identify it and adjust its open probability. Among other SUR regions that interacts with Kir6.2[10], one of the transmembrane domains of SUR, TMD0, is known to tightly bind to Kir6.2 and to facilitate its trafficking to the plasma membrane[11,12].

Here, we report the successful engineering and characterization of β2-based ICCRs. A prerequisite to this project was to find a way to overcome poor surface expression of β2AR-Kir6.2 fusion proteins. This was achieved by co-expression of TMD0 of isoform SUR1 which dramatically increased surface expression of all constructs through its interactions with Kir6.2. Pharmacological characterization of β2 ICCRs demonstrated concentration-dependent effects of β-adrenergic agonists and antagonists. In addition, the amplitude of the agonist-induced signal depended on the receptor-channel linker length, corroborating previous observations[1] and demonstrating the crucial role of the receptor C-terminus in coupling efficiency. We also examined the effect of a β2AR stabilizing mutation at position 3.41 in the Ballesteros/Weinstein scheme[13,14] on the communication between receptor and channel and found that it logically reduced the amplitude of the agonist responses.

Part of this work has been published in abstract form[15].

### Results

#### Design of β2 ICCRs

Building the original M2 and D2 ICCRs helped delineate the blueprints for other ICCRs. We therefore used the M2 and D2 ICCRs as templates for expedient design of β2 ICCRs. Although M2 and D2 display a low overall sequence similarity of <30% with β2AR, the sequence of the H8 helix is well conserved and was used to unambiguously align the C-terminal extremities of the receptors (Fig.1). This alignment shows that the C-terminus of β2AR is much longer than that of M2 and D2. Reasoning that this long C‑terminus might preclude proper coupling, we constructed three β2‑based ICCRs: one using the full-length receptor, and two comprised of C­terminal truncated forms of β2AR (β2ΔC62 and β2ΔC72), equivalent in length to the M2 and D2 receptors, respectively. These receptors were fused to a truncated Kir6.2 lacking its first 25 N-terminal residues, a modification that was shown to produce the most efficient coupling in M2 and D2 ICCRs[1]. To designate the constructs, we use the nomenclature R-K-X-Y, where R is replaced by the short name of the receptor, K stands for Kir6.2, X and Y are the number of residues removed from the receptor C-terminus and channel N-terminus, respectively. The β2 constructs are therefore named β2‑K0‑25, β2‑K‑62‑25, and β2‑K‑72‑25.

#### Optimizing surface expression

Constructs were expressed in *Xenopus* oocytes and characterized by the two-electrode voltage clamp technique. As a rough estimate of surface expression levels, we measured the basal currents, i.e., the initial whole-cell currents, (Fig. 2). The three β2 constructs produced basal currents that were equivalent to those obtained with non-injected oocytes suggesting no or little expression of active channels. In an attempt to solve this expression problem, we engineered ICCRs using the β2(E122W) mutant. This mutation of Glu122 to Trp122 at Ballesteros/Weinstein position 3.41[13] has been shown to enhance the surface expression level of the β2-adrenergic receptorin insect and mammalian cells by stabilizing the TM4-TM3-TM5 helix interface[14]. This mutation had no effect on the basal current of β2-based ICCRs.

It has been demonstrated that N-terminal deletions could favor cell surface expression of the cannabinoid receptor 1[16] and the α1D-adrenergic receptor[17]. We therefore tried gradual N-terminal deletions of the first 10 to 25 residues of β2AR in construction β2‑K‑62‑25. The data shown in Fig. S1 show that these modifications did not improve expression. Also shown in Fig. S1 are the disappointing outcomes of using N-terminal and C-terminal chimera between β2AR and the robustly-expressed M2 receptor.

We then tested the co-expression of TMD0, a 195-residue N-terminal transmembrane domain of SUR1, known to facilitate Kir6.2 trafficking[11], with β2-based ICCRs. The resulting basal current was increased 5-fold for β2‑K0‑25 and β2‑K‑62‑25 and 7-fold for β2‑K‑72‑25 compared to the ICCRs expressed alone. Thus, we found an efficient way to enhance significantly surface expression levels of the β2-based ICCRs. These results suggest that TMD0 helps the β2-based ICCRs reach the membrane because of its chaperone role on Kir6.2.

#### Demonstration of direct receptor-channel coupling

The functionality of the coupling between β2AR(full-length, ΔC62, ΔC72) and Kir6.2 was tested with the β-adrenergicagonist isoproterenol. We initially verified that isoproterenol had no direct or receptor-mediated effects on Kir6.2 alone or co-expressed with β2AR (Fig. 3B). When the fusion proteins where expressed (with TMD0), β2‑K0‑25 did not respond to isoproterenol whereas β2‑K‑62‑25 and β2‑K‑72‑25 were strongly activated (Fig. 3). Isoproterenol responses were concentration-dependent with no obvious cooperativity (Hill coefficients ~1). Given the variability in the data, the EC50 of 149 nM for β2‑K‑62‑25 and 288 nM for β2‑K‑72‑25 were not significantly different (p=0.31; unpaired Student's t-test). These values are consistent with those from other techniques that do not rely on G-protein signalling such as competitive radioligand binding or fluorescence spectroscopy[18,19]. The maximal channel activation was 64% of the basal current for β2‑K‑62‑25 and 37% for β2‑K‑72‑25, a statistically significant difference (p=0.018). This change in efficacy without change in affinity underscores the role of the length of the receptor-channel linker region in efficient transmission of the ligand-induced β2 conformational change to the channel gate.

We next tested the effect of the antagonist alprenolol at 5 µM on the isoproterenol-activated current. Alprenolol did not alter the current generated by the isoproterenol-insensitive construct β2‑K0‑25 but it caused a complete block of isoproterenol activation of β2‑K‑62‑25 and β2‑K‑72‑25 (Fig. 4). This block could not be washed out after several minutes, probably because we used a relatively high alprenolol concentration. These results confirmed the specificity of isoproterenol effect on the β2 adrenergic receptor.

#### Partial and inverse agonists

Full agonists can cause maximal activation of the receptor whereas partial agonists cause an activation which remains less than maximal even at saturating concentrations. It is thought that full and partial agonists of the β-adrenoceptor do not trigger the same conformational changes in the receptor. As a comparison with the full agonist isoproterenol, we therefore assayed the partial agonist salbutamol on construct β2‑K‑62‑25. As shown in Fig. 5, salbutamol strongly activated β2‑K‑62‑25. The maximal activation, 78% of the basal current at 50 µM, was larger, though not significantly (p=0.12) than that achieved by isoproterenol, 63% at 50 µM. The concentration dependence was not as steep with a Hill slope of 0.64 compared to 1.04 for isoproterenol. Although these differences remain rather subtle, they reinforce the notion of distinct modes of action for partial and full agonists[20].

Inverse agonists, thought to target the agonist binding site, downregulates the receptor by blocking its constitutive activity. We examined the effects of the inverse agonist timolol[21]. At concentrations up to 50 µM, timolol did not produce any significant change in the electrical signal from either β2‑K‑62‑25 or β2‑K‑72‑25 (Fig.5C). This lack of effects suggests that binding of timolol does not induce a large conformational change in the receptor.

Another possibility could be that the ICCRs are partly cleaved and that we could have an unresponsive Kir6.2 breakdown product responsible for the high basal current together with a responsive full-length fusion construct with no basal current. In that case, an already inactive construct could not possibly be further inhibited by timolol. This hypothesis is highly improbable because 1) we have never detected any breakdown products by Western blot in other similar fusion constructs not included in the present work, and 2) Fig. S2 shows that Kir6.2 + TMD0 produces a basal current that is barely detectable.

#### A stabilizing mutation alters coupling

To further show that ICCR systems can be used as a functional characterization tool, we set out a study on the E122W β2AR mutant described above. As described in Fig. 6, for construct β2‑K‑62‑25, mutation E122W appeared to reduce the amplitude of the agonist-induced signal (from 63.5% to 51% at maximum activation) and to increase dissociation constant (from 149 nM to 247 nM) but these effects did not reach statistical significance (p=0.12 for amplitudes; p=0.24 for affinities). The mutation had a stronger effect on construct β2‑K‑72‑25 since maximal activation decreased from 37% to 11%, a statistically significant change (p=0.017). In that case, affinities could not be compared because the activation of the β2(E122W)‑K‑72‑25 was too weak for proper fitting. These results could be explained by the fact that stabilization of the TM4-TM3-TM5 helix interface[14] induces less important conformational change in β2AR upon ligand binding. Indeed, position 3.41 is located at the TM4-TM3-TM5 interface and the Trp ring may interact with Pro2115.50 partially decreasing TM5 flexibility. Since TM5 is assumed to serve as an intermediate between the TM1-4 structural core and TM6-7[22], we can imagine that the quality of the transmission of conformational change may be constrained by such mutation. This might lead to a less efficient communication with Kir6.2 resulting in decreased amplitude of the response.

### Discussion

Using the ICCR concept established with M2 and D2 receptors[1], we have used the β2-adrenergic receptor to create synthetic ligand-gated K+ channels sensitive to β-adrenergic ligands.

#### Surface expression enhancement by an accessory Kir6.2-binding protein

A recurrent difficulty with recombinant membrane proteins is the low density of proteins that reach the plasma membrane. Although *Xenopus* oocytes are very tolerant in that respect, expression of the β2-Kir6.2 fusion constructs produced no discernible electrophysiological signals. Suspecting a trafficking impediment, we searched for ways to enhance surface expression. It is known that Kir6.2 possess a C-terminal endoplasmic-reticulum retention signal[23] but removal of this signal in M2 ICCRs did not augment surface expression[1]. The mutation E122W in β2AR, reported to increase surface expression[14] was also not beneficial. The solution came from the KATP channel. That channel is a complex of Kir6.2 and the protein SUR. Association of SUR to Kir6.2 is known to be mediated in large part by its N-terminal transmembrane domain TMD0, a ~200-residue alpha-helical region that binds to Kir6.2 by itself and can promote its targeting to the surface membrane[11]. When the TMD0 domain of the sulfonylurea receptor isoform SUR1 was co-expressed with the various β2-Kir6.2 constructs, large K+ currents could be recorded indicative of the presence of active Kir6.2 at the oocyte surface. This discovery was the key to the pursuit of the project. It suggests that, in the tetrameric β2-Kir6.2 complexes, there is ample space for TMD0 to bind to Kir6.2 and to act as a chaperone to promote proper membrane targeting.

#### Functional β2 ICCRs

The β2 ICCRs were engineered by covalent linkage of β2AR to the Kir6.2 channel to promote physical interactions between the two proteins. Functional coupling could only be achieved after removal of 25 residues from the Kir6.2 N-terminus, as in previous ICCRs, and also of 62 to 72 residues from the β2AR C-terminus whereas M2 and D2 ICCRs used unmodified receptors. These residues which are not resolved in crystallographic structures[4] probably form flexible elements[24] that dampen transmission of mechanical perturbations from receptor to channel. The dependence of responses on the length of the receptor-channel argues strongly for a direct, physical interaction between receptor and channel. We also verified the lack of detectable G-protein dependent modulation of Kir6.2 by β2AR in control experiments where receptor and channel were coexpressed as separate proteins. Furthermore, β2AR is predominantly Gs-coupled, the M2 receptor is Gi-coupled, but both produce similar effects when fused to Kir6.2.

#### ICCRs as conformational motion detectors

Constructs β2‑K‑62‑25 and β2‑K‑72‑25 (+TMD0) detected the presence of agonists with dose-dependent correlation, in direct, real-time and label-free conditions. The affinity measured for the full agonist isoproterenol matched those obtained by radioligand assays[14,19] as well as spectroscopy assays that, like ICCRs, directly measure conformational changes[18]. The effect of the partial agonist salbutamol was similar to that of isoproterenol although it showed lower affinity as expected. Isoproterenol and salbutamol have been shown to induce distinct conformations. In particular, evidence suggests that both disrupt the cytoplasmic ionic lock while only isoproterenol uses the rotamer toggle switch[25]. The similarity of the responses elicited by salbutamol and isoproterenol suggests that the conformational changes detected by the channel could be related to the ionic lock rather than the rotamer toggle switch[25]. Because by construction ICCRs report on the motion of the GPCR C-terminus, this would imply that disruption of the ionic lock triggers a conformational change in the C-terminus.

The effect of the antagonist alprenolol was easily detectable by abolition of the agonist-induced increase of the ionic current. If alprenolol did not change basal signal, inverse agonists are expected to reduce basal activity and elicit signals in absence of agonists. In the ICCR assay, the inverse agonist timolol produced no significant signal. Although this observation could result from an intrinsically low basal activity of β2AR due to the expression system or the fusion to Kir6.2, it shows that binding of timolol does not induce any detectable conformational change of the C-terminus. Such conclusion is consistent with a recent crystallographic study[26] showing only very small differences between the antagonist-bound and inverse-agonist-bound structures of β2AR.

Thus, beside the obvious use of ICCRs in drug screening, they could be valuable to dissect the conformational changes induced by ligands. We provided an additional example of such use by demonstrating that a stabilizing mutation, E122W3.41[14], reduced the amplitude of the ICCR response in line with its purported attenuation of conformational changes.

#### Physiological relevance

ICCRs demonstrate that, provided a GPCR is tightly associated with an ion channel, it can directly modulate channel gating possibly through mechanical forces transmitted by its C-terminal tail. Did evolution overlook this seemingly trivial possibility of using localized modulation in addition to the more indiscriminate second-messenger pathways? Probably not, as there is solid evidence that receptors and channels can form stable complex[27-29]. Channel modulation via the C-terminal tail of GPCRs has been reported for 2 couples, GABAA channel/dopamine D5 receptor[30] and NMDA channel/dopamine D1 receptor[31]. ICCRs could provide a model for these interactions as well as for others involving Kir channels[32].

#### β-adrenergic ligand-activated K+ channels

Like traditional ligand-gated channels such as the cationic nicotinic acetylcholine receptor or the anionic GABAA receptor[33], β2 ICCRs incorporates in a single polypeptide chain a binding site for a specific signaling molecule and an ion-selective pore that are allosterically linked. They possess, however, the unique features among ligand-gated channels of being activated by β‑adrenergic signals and of being selective for potassium ions. One may envision that these ICCRs could be used as novel regulatory elements in synthetic biology as well as therapeutic tools. Such use is of course remote and would require to augment trafficking efficiency to avoid using accessory proteins such as TMD0 and optimize response efficacy so that channels are closed at rest and open upon stimulation like existing ligand-gated channels. This would require protein engineering that is now complex but could become more straightforward as determinants of membrane protein trafficking and of channel gating are clarified.

### Materials and Methods

Molecular biology

Experiments were conducted as previously described[1]. In this work, we used mouse Kir6.2 (Genbank D50581)[34], human β2-adrenergic receptor (Genbank NM_000024.3), hamster TMD0(SUR1)-F195[11,35], mouse Kir6.2ΔC36[36]. The β2‑K0-25 fusion was obtained by replacing the muscarinic M2 receptor gene in M2‑K0‑25 cloned in the *Xenopus* oocyte expression vector pGEMHE[1]. Insertion of the β2AR gene and deletion of the M2 gene was performed using a two-step PCR. In the first PCR reaction, the β2-adrenergic gene was amplified from its original pCMV vector using hybrid primers complementary to the β2‑adrenergic sequence 3' extremities and to the flanking regions of the insertion site in the M2‑Kir6.2_ pGEMHE. The products of this reaction were gel-purified (QIAquick Gel Extraction Kit, Qiagen) and served as primers for a second PCR with M2-K0-25 as a template, yielding β2‑K0‑25_pGEMHE. Alignments of the M2, D2, and β2 receptor sequences with ClustalX[37] were adjusted manually to position conserved helix H8. The unstructured C-terminal region downstream of H8 was longer in the β2AR by 62 and 72 amino acids compared to M2 and D2, respectively (Fig. 1). To match the lengths of M2 and D2, additional β2-K constructs with shorter β2AR C-termini were obtained in a single-step PCR using the β2‑K0‑25 construct as a template and hybrid oligonucleotides flanking the deleted region[38]. Mutation E122W was introduced in each ICCR in a single-step PCR with oligonucleotides incorporating the mutation. Reagents and conditions were from the QuikChange site-directed mutagenesis kit (Agilent Technologies). Positive clones were identified by restriction enzyme profiling and verified by sequencing the full open reading frame.

After DNA amplification, constructs were linearized and mRNAs synthesized using the T7 mMessage mMachine Kit (Ambion). mRNAs were purified either by standard phenol:chloroform extraction or using the MEGAclear Purification Kit (Ambion), and quantified by agarose-gel electrophoresis and spectrophotometry.

Electrophysiological recordings

Animal handling and experiments fully conformed with French regulations and were approved by local governmental veterinary services (authorization no. 38-08-10 from the Ministère de l’Agriculture, Direction des Services Vétérinaires to Michel Vivaudou). Oocytes were surgically removed from *Xenopus laevis* and defolliculated by three 30 min-incubations in 2 mg.ml-1 type 1A collagenase solution at 19°C. Stage V and VI oocytes were microinjected with 50 nl of RNase-free water containing one or a mixture of the following quantities of RNA: β2-Kir6.2, 5 ng; Kir6.2ΔC36, 2 ng; TMD0(SUR1)-F195, 1 ng. Microinjected oocytes were incubated for >2 days at 19°C in Barth’s solution (in mM: 1 KCl, 0.82 MgSO4, 88 NaCl, 2.4 NaHCO3, 0.41 CaCl2, 16 Hepes, pH 7.4) supplemented with 100 U.ml-1 penicillin, streptomycin and gentamycin. All chemicals were purchased from Sigma-Aldrich. Whole-cell currents were recorded with the two-electrode voltage clamp (TEVC) technique using a GeneClamp 500 amplifier (Molecular Devices). Microelectrodes were filled with 3 M KCl and oocytes were bathed in the following solution (in mM): 91 KCl, 1.8 CaCl2, 1 MgCl2, 5 HEPES, 0.3 niflumic acid (to block endogenous Cl- currents), pH 7.4. The TEVC voltage protocol consisted of 500-ms steps to ‑50, 0 and +50 mV  during which current was measured  separated by 5 s at a holding potential of 0 mV. The values shown in the figures are those recorded at -50 mV.

Data analysis

Basal current was measured while oocytes were in standard bath solution during the first minute of recording. Ba2+ (3 mM) was used as a generic potassium-channel blocker to establish the amount of exogenous current, designated as Ba2+-sensitive current and calculated by subtracting from all measured values the value measured at the end of an experiment after application of 3 mM Ba2+. All values of current reported here refer to Ba2+-sensitive currents. Changes in Ba2+-sensitive currents by effectors were calculated with respect to the value measured before application. The points at which the current were measured on the current traces are indicated by arrows in the figures. For the concentration-response data, obtained by sequential application of increasing agonist concentrations, changes in current were calculated only with respect to the current before application of the initial, lowest concentration.

Average values are presented as mean±s.e.m. Non-linear least-square curve-fitting was carried out with Origin 8 software (OriginLab) using a standard Hill equation:

f(x) = Max / [ 1 + (EC50 / x)h ]

where x is the concentration of a ligand, Max the asymptotical maximal effect, EC50 the concentration for half-maximal effect, and h the Hill coefficient. The fits shown in the figures were performed using average data. For statistical analysis of parameters Max and EC50 (using Origin 8 software), individual dose-response data from each oocyte tested were fitted using the above equation with h=1 to obtain a set of values of Max and EC50 for each construct and ligand. Statistical significance for these parameters and for other experimental data was established with unpaired two-tailed Student t-tests and is indicated as p-values in the text.

## Acknowledgments

We are grateful to S. Seino (Chiba, Japan) for mouse Kir6.2 and K. Chan (Cleveland, OH) for the construct TMD0(SUR1).

## References

1. Moreau CJ, Dupuis JP, Revilloud J, Arumugam K, Vivaudou M (2008) Coupling ion channels to receptors for biomolecule sensing. Nature Nanotech. 3: 620-5.

2. Minneman KP, Pittman RN, Molinoff PB (1981) Beta-adrenergic receptor subtypes: Properties, distribution, and regulation. Annu Rev Neurosci. 4: 419-61.

3. Frishman W (2008) beta-Adrenergic blockers: a 50-year historical perspective. Am J Ther. 15: 565-76.

4. Cherezov V, Rosenbaum DM, Hanson MA, Rasmussen SG, Thian FS et al. (2007) High-resolution crystal structure of an engineered human ß2-adrenergic G protein-coupled receptor. Science. 318: 1258-65.

5. Moreau C, Prost AL, Derand R, Vivaudou M (2005) SUR, ABC proteins targeted by KATP channel openers. J Mol Cell Cardiol. 38: 951-63.

6. Mikhailov MV, Campbell JD, de Wet H, Shimomura K, Zadek B et al. (2005) 3-D structural and functional characterization of the purified KATP channel complex Kir6.2-SUR1. EMBO J. 24: 4166-75.

7. Moreau C, Jacquet H, Prost AL, D'Hahan N, Vivaudou M (2000) The molecular basis of the specificity of action of KATP channel openers. EMBO J. 19: 6644-51.

8. Nichols CG (2006) KATP channels as molecular sensors of cellular metabolism. Nature. 440: 470-6.

9. Antcliff JF, Haider S, Proks P, Sansom MS, Ashcroft FM (2005) Functional analysis of a structural model of the ATP-binding site of the KATP channel Kir6.2 subunit. EMBO J. 24: 229-39.

10. Dupuis JP, Revilloud J, Moreau CJ, Vivaudou M (2008) Three C-terminal residues from the sulphonylurea receptor contribute to the functional coupling between the KATP channel subunits SUR2A and Kir6.2. J Physiol. 586: 3075-85.

11. Chan KW, Zhang H, Logothetis DE (2003) N-terminal transmembrane domain of the SUR controls trafficking and gating of Kir6 channel subunits. EMBO J. 22: 3833-43.

12. Hosy E, Derand R, Revilloud J, Vivaudou M (2007) Remodelling of the SUR-Kir6.2 interface of the KATP channel upon ATP binding revealed by the conformational blocker rhodamine 123. J Physiol. 582: 27-39.

13. Ballesteros JA, Weinstein H (1995) Integrated methods for the construction of three dimensional models and computational probing of structure-function relations in G-protein coupled receptors. Methods Neurosci. 25: 366-428.

14. Roth CB, Hanson MA, Stevens RC (2008) Stabilization of the human beta2-adrenergic receptor TM4-TM3-TM5 helix interface by mutagenesis of Glu122(3.41), a critical residue in GPCR structure. J Mol Biol. 376: 1305-19.

15. Caro LN, Moreau CJ, Revilloud J, Dupuis JP, Vivaudou M (2010) Design of Biosensors Based on the Covalent Assembly of G-Protein Coupled Receptors and Potassium Channels. Biophys J. 98: 193a.

16. Andersson H, D'Antona AM, Kendall DA, Von Heijne G, Chin CN (2003) Membrane assembly of the cannabinoid receptor 1: impact of a long N-terminal tail. Mol Pharmacol. 64: 570-7.

17. Hague C, Chen Z, Pupo A, Schulte N, Toews M et al. (2004) The N terminus of the human alpha1D-adrenergic receptor prevents cell surface expression. J Pharmacol Exp Ther. 309: 388-97.

18. Yao X, Parnot C, Deupi X, Ratnala VR, Swaminath G et al. (2006) Coupling ligand structure to specific conformational switches in the beta2-adrenoceptor. Nat Chem Biol. 2: 417-22.

19. Baker J (2010) The selectivity of beta-adrenoceptor agonists at human beta1-, beta2- and beta3-adrenoceptors. Br J Pharmacol. 160: 1048-61.

20. Rosenbaum DM, Rasmussen SG, Kobilka BK (2009) The structure and function of G-protein-coupled receptors. Nature. 459: 356-63.

21. Taira C, Monczor F, Hocht C (2010) Measurement of inverse agonism in beta-adrenoceptors. Methods Enzymol. 485: 37-60.

22. Chelikani P, Hornak V, Eilers M, Reeves PJ, Smith SO et al. (2007) Role of group-conserved residues in the helical core of beta2-adrenergic receptor. Proc Natl Acad Sci U S A. 104: 7027-32.

23. Zerangue N, Schwappach B, Jan YN, Jan LY (1999) A new ER trafficking signal regulates the subunit stoichiometry of plasma membrane KATP channels. Neuron. 22: 537-48.

24. Granier S, Kim S, Shafer A, Ratnala V, Fung J et al. (2007) Structure and conformational changes in the C-terminal domain of the beta2-adrenoceptor: insights from fluorescence resonance energy transfer studies. J Biol Chem. 282: 13895-905.

25. Kobilka BK, Deupi X (2007) Conformational complexity of G-protein-coupled receptors. Trends Pharmacol Sci. 28: 397-406.

26. Wacker D, Fenalti G, Brown M, Katritch V, Abagyan R et al. (2010) Conserved binding mode of human beta2 adrenergic receptor inverse agonists and antagonist revealed by X-ray crystallography. J Am Chem Soc. 132: 11443-5.

27. Davare MA, Avdonin V, Hall DD, Peden EM, Burette A et al. (2001) A beta2 adrenergic receptor signaling complex assembled with the Ca2+ channel Cav1.2. Science. 293: 98-101.

28. Lavine N, Ethier N, Oak JN, Pei L, Liu F et al. (2002) G protein-coupled receptors form stable complexes with inwardly rectifying potassium channels and adenylyl cyclase. J Biol Chem. 277: 46010-9.

29. Kisilevsky AE, Mulligan SJ, Altier C, Iftinca MC, Varela D et al. (2008) D1 Receptors Physically Interact with N-Type Calcium Channels to Regulate Channel Distribution and Dendritic Calcium Entry. Neuron. 58: 557-70.

30. Liu F, Wan Q, Pristupa ZB, Yu XM, Wang YT et al. (2000) Direct protein-protein coupling enables cross-talk between dopamine D5 and γ-aminobutyric acid A receptors. Nature. 403: 274-80.

31. Lee FJ, Xue S, Pei L, Vukusic B, Chery N et al. (2002) Dual regulation of NMDA receptor functions by direct protein-protein interactions with the dopamine D1 receptor. Cell. 111: 219-30.

32. Doupnik CA (2008) GPCR-Kir channel signaling complexes: Defining rules of engagement. J Recept Signal Transduct Res. 28: 83-91.

33. Corringer PJ, Baaden M, Bocquet N, Delarue M, Dufresne V et al. (2010) Atomic structure and dynamics of pentameric ligand-gated ion channels: new insight from bacterial homologues. J Physiol. 588: 565-72.

34. Inagaki N, Gonoi T, Clement JP, Namba N, Inazawa J et al. (1995) Reconstitution of I-KATP: An inward rectifier subunit plus the sulfonylurea receptor. Science. 270: 1166-70.

35. Hosy E, Dupuis JP, Vivaudou M (2010) Impact of disease-causing SUR1 mutations on the KATP channel subunit interface probed with a rhodamine protection assay. J Biol Chem. 285: 3084-91.

36. Tucker SJ, Gribble FM, Zhao C, Trapp S, Ashcroft FM (1997) Truncation of Kir6.2 produces ATP-sensitive K+ channels in the absence of the sulphonylurea receptor. Nature. 387: 179-83.

37. Thompson JD, Gibson TJ, Plewniak F, Jeanmougin F, Higgins DG (1997) The CLUSTAL_X windows interface: flexible strategies for multiple sequence alignment aided by quality analysis tools. Nucleic Acids Res. 25: 4876-82.

38. Makarova O, Kamberov E, Margolis B (2000) Generation of deletion and point mutations with one primer in a single cloning step. Biotechniques. 29: 970-2.

## Figure Legends

**Fig. 1. Design strategy of β2-based Ion Channel-Coupled Receptors.** ICCRs were formed by covalent linkage of GPCRs C-termini to Kir6.2 channel N-terminus. Helix H8 and β-bridge β1 are predicted from the β2AR (PDB code: 2RH1) and chimeric Kir3.1 (PDB code: 2QKS) structures, respectively. M2-K0-25 and D2-K0-25 are the ICCRs previously shown to be functional with 25 residues deleted from the Kir6.2 N-terminus. We used the same Kir6.2 deletion to build β2 ICCRs, with additional deletions in the receptor C-terminus. β2-K0-25 ICCR contains the full-length receptor, β2-K-62-25 and β2-K-72-25 are based on the β2AR deleted of 62 and 72 residues in its C-terminal domain to match the lengths of M2 and D2, respectively.

**Fig. 2. TMD0 of SUR boosts expression of β2-based ICCRs.** Basal currents are the whole-cell currents measured in the first minute of TEVC recording from unstimulated *Xenopus* oocytes. E122W is a mutation of residue 122 of β2AR from Glu to Trp reported to increase β2 surface expression.TMD0 is the first transmembrane domain of the sulfonylurea receptor SUR1, a physiological partner of Kir6.2. *P<0.05 and **P<0.00001 represent significant differences from the basal current measured in non-injected oocytes.

**Fig. 3. Receptor-channel coupling in β2 ICCRs: response to the agonist isoproterenol.** (*A*) Representative TEVC recordings from *Xenopus* oocytes expressing each β2 ICCR and TMD0. Membrane potential was -50 mV. Dashed lines indicate the baseline of Ba2+-sensitive currents. (*B*) Concentration-effect curves for isoproterenol measured in oocytes co-expressing the indicated proteins. Kir6.2ΔC36 is deleted of its last 36 residues to allow surface expression of the channel alone. Values are average of 5-14 measurements. Smooth lines correspond to Hill equations fits with EC50 in parentheses and h=1.07 for β2‑K‑62‑25 and 1 for β2-­K-­72-­25.

**Fig. 4. Effect of a β-adrenergic antagonist on β2 ICCRs.** (*A*) TEVC recordings showing antagonist effect of 5 µM alprenolol during addition of 0.5 µM isoproterenol on β2-K0-25, β2-K-62-25 and β2-K-72-25. (*B*) Change in whole-cell currents evoked by isoproterenol before and after addition of 5 µM alprenolol. **P<0.00075 indicates a significant inhibition induced by alprenolol.

**Fig. 5. Effect of a β2AR partial agonist on β2-K-62-25.** Concentration-effect curves for salbutamol measured in oocytes co-expressing the indicated proteins. Values are average of 3-7 measurements. The smooth line is a Hill equation fit to the β2-K-62-25+TMD0 data with EC50 = 452 nM and h = 0.6. Data obtained with the unfused Kir6.2 as a control could not be fitted.

**Fig. 6. The stabilizing mutation E122W weakens agonist-induced channel responses.** (*A*) Location of Glu122 (in red) in the β2-adrenergic receptor structure. (*B*) Concentration-effect curves of isoproterenol on β2-K-62-25 and β2-K-72-25, unmodified (WT) and harboring mutation E122W (all co-expressed with TMD0). Values are average of 5-14 measurements. Hill equation fits, represented as smooth lines, yielded EC50 of 149 nM, 247 nM, and 288 nM for β2-K-62-25, β2(E122W)-K-62-25, and β2‑K‑72‑25, respectively. h was 1.07, 1, and 1.18.

**Fig. S1.** Expression levels of various β2-K-62-25 constructs designed in an attempt to improve surface expression. The basal currents, whole-oocyte currents recorded in absence of agonist are taken as an indicator of the number of active channels at the cell surface. ΔN10, ΔN15, ΔN20, and ΔN25 designate constructs based on β2-K-62-25 with the first N-terminal 10, 15, 20, and 25 residues of ß2AR deleted. Nt(M2)ΔN28 is a β2-K-62-25 chimera where the extracellular N-terminal of ß2AR (28 residues) has been replaced by that of the M2 receptor (18 residues). Ct(M2) is a β2-K-62-25 where the intracellular C-terminal of β2ARΔC62 (residues 326 to 352) has been replaced by that of the M2 receptor (residues 440 to 466).

**Fig. S2.** Comparison of the expression levels of Kir6.2, alone or fused to β2, coexpressed with TMD0. The basal currents, whole-oocyte currents recorded in absence of agonist are taken as an indicator of the number of active channels at the cell surface.
